# Chronic General Diseases, or Constitutional Affections; Their Causes, Pathology and the Principles That Should Guide Their Treatment

**Published:** 1882-09

**Authors:** N. S. Davis

**Affiliations:** Chicago, Ill.


CHICAGO MEDICAL

### JOURNAL & EXAMINER.

VOL. XLV.—SEPTEMBER, 1882.—NO. 3.

### Original Lectures.

ARTICLE I.

### CHRONIC GENERAL DISEASES, OR CONSIITUTIONAL AFFECTIONS: THEIR CAUSES, PATHOLOGY, AND THE PRINCIPLES THAT SHOULD GUIDE THEIR TREATMENT. A Lecture in the Regular Course on Practical Medicine in the Chicago Medical College. By N. S. DAVIS, M. D., Chicago, Ill.

*Gentlemen:* Having completed the consideration of the acute general diseases, I now invite your attention to the second division of general diseases called chronic or constitutional affections. Under this head belong scrofula, tuberculosis, leucocythæmia. pernicious anæmia, Addison's disease, carcinoma, constitutional syphilis, rheumatism and gout.

Diverse from each other as some of these diseases may appear to be, they nevertheless have a sufficient number of circumstances in common to justify their being grouped together.

First. They are all characterized by a very persistent, if not permanent, alteration of the natural properties of the tissues, giving rise to certain morbid tendencies or predispositions to the development of special local affections both of a functional and structural character.

Second. They are all capable of being transmitted from parent to child—in other words, of being perpetuated by hereditary influence.

Third. So far as relates to the general or constitutional morbid condition, there is no tendency to a self-limited duration.

Fourth. They all arise from causes acting with feeble intensity, but persistently, through long periods of time, and of such a nature as to modify one or both of the elementary properties of living, organized matter.

In all these particulars, this group of constitutional diseases stands in direct contrast with the class of acute general diseases which we have already passed in review. You have observed that, in all the latter, the morbid manifestations are of an active character, leading rapidly to functional and structural changes of limited duration, are incapable of hereditary transmission, and arise from causes acting with more intensity, but of limited duration, one full impression of which often destroys the susceptibility to any further action of the same cause. On the contrary, the diseases I am about to discuss are based on such changes in the properties of the primary organic molecules entering into the various structures of the body, as give such molecules certain tendencies to deviate from the natural standard or type of development, leading, if not counteracted by adverse influences, sooner or later, to such alterations in the molecular movements constituting nutrition and disintegration as to develop structural changes, consisting of local hypertrophies, atrophies, tissue degenerations, or morbid growths, according to the degree and direction of the primary deviations.

So slight and occult are the original changes in the properties of the organic atoms or cells that the constitutional vice or defect may exist for years without any appreciable structural changes, as we see in those hereditarily predisposed to pulmonary tuberculosis, carcinoma, etc., and yet if at any time during the life of such individuals, ordinary exciting causes chance to induce local irritation or inflammation, the presence of the latent or constitutional condition is made manifest by the unusual persistence of the local morbid action and the special tendency to degenerative changes in the exudations or other products resulting therefrom. For instance, a child possessing the scrofulous diathesis or constitutional condition, if exposed to a current of cold air upon the neck, may have inflammation, exudation, and tumefaction of the lymphatic glands, ending either in permanent hypertrophy, caseous degeneration, or destructive suppuration; when the same cause provoking a similar degree of inflammation in a strictly healthy child, would have caused but a temporary exudation and swelling, to be followed in a few days by resolution and a return to the natural condition. So an adult with the tuberculous diathesis or constitutional condition, attacked with pneumonia followed by the usual exudation, will be likely to have such exudative material undergo either purulent degeneration, constituting diffuse suppuration, or caseous degeneration and early phthisis, instead of resolution and re-absorption, as usual in subjects previously healthy.

That the primary and essential pathological condition constituting a chronic general disease, constitutional vice, cachexia, or diathesis, as it is variously called by different authors, consists in a morbid condition of one or both of the inherent properties of organized living matter, is proved both by its liability to hereditary transmission, and its persistence indefinitely with a well-known tendency to develop, sooner or later, specific nutritive changes in some of the structures of the body. As the germinal cell or aggregation of bioplasm furnished by the female, and the spermatozoa furnished by the male, are both living organized materials, it is reasonable to suppose that they will partake of the same properties, whether perfect or imperfect, that belong to all the other organized atoms of the bodies in which they were developed; and consequently, in their independent subsequent growth, they will generally develop the same morbid tendencies as were possessed by the parent. The modifications of the properties of the germ may be so strong as to lead to manifest errors of nutrition during the development of the fœtus in utero, or at any time during the period of subsequent growth, or so feeble as not to cause their appearance until after the climax of adult life in the early stage of physical decline. While the essential pathology of all this class of diseases consists in some modification of the elementary properties that govern the molecular changes constituting nutrition and growth, these modifications not only differ in degree in different cases in the same constitutional affection, but they also differ in kind or direction in each affection from all the others; so that the local manifestations of disease developed from time to time during the progress of any given case, are peculiar to the special constitutional affection to which the case belongs.

For instance, the general morbid condition constituting scrofula, never gives rise to the local, functional or structural changes characteristic of syphilis, carcinoma, or leucocythæmia, and *vice versa.* Neither do you find the rheumatic constitution giving rise to the local inflammations of gout, or the reverse. This affords further proof that the primary or fundamental pathological condition consists in some deviation from the natural condition of the properties inhering in the organized tissue elements, inasmuch as it shows obedience to the universal law of living matter, namely, that like begets like. It is true, that constitutional syphilis may be established in a subject already scrofulous, or by a sufficient exposure to the proper causes and modes of living, gouty affections may be engrafted upon a previously rheumatic diathesis; but this in no proper sense invalidates the law just stated in regard to the fixed tendencies of each constitutional disease. Another fact of much pathological importance is, that the local affections which are liable to appear during the unrestrained progress of any one of the general diseases included in the class under consideration, are not accidental complications, but natural or necessary outgrowths resulting from the progress of the constitutional vice. They may be hastened in their appearance, or rendered more severe by the intervention of special exciting causes, or the influence of bad sanitary conditions. And, on the other hand, their development may be retarded or entirely prevented by the combined influence of good climatic, hygienic, and sanitary regulations.

And yet, in a large majority of cases, it is the local morbid developments that chiefly occupy the attention of the patient, and on account of which he seeks the aid of his physician. The local developments resulting from the progress of the scrofulous diathesis, appear most frequently in some part of the adenoid or lymphatic glandular system, and next in the cutaneous surface. Those of the tuberculous, which is closely allied to, if not a mere modification of the scrofulous, may be met with in any of the more vascular structures of the body, but are most frequent in the lungs, and next in the mucous membranes and lymphatic glands. In leucocythæmia and pseudo-leucocythæmia, the almost uniform tendency is to develop hypertrophy or increased growth of the lymphatic glands and spleen.

*Etiology.*—There is no doubt that a large proportion of the cases of the several diseases included in this group have their origin primarily in hereditary influence. This I have endeavored to explain already; but cases are also met with in relation to which no hereditary influence can be traced. These appear to have had their origin from certain causes which had been permitted to act steadily through long periods of time, and yet with so moderate a degree of intensity as to avoid exciting acute general disturbances. One class of these causes produce their deleterious effects by acting primarily on the processes of digestion, assimilation and nutrition; another class exert their influence on the processes of disintegration and elimination of waste material. To the first, belong insufficient or unwholesome food; inadequate supply of light, heat, and pure air, and want of proper exercise. To the latter, belong all those agents and influences that slowly but persistently retard retrograde metamorphosis in the tissues, or interfere with the elimination of the products of such metamorphosis through the proper excretory organs, such as continued exposure to cold and damp air; deficient physical exercise; depressing mental emotions; the habitual use of alcoholic drinks; and the occupation of rooms overcrowded, or inadequately supplied with air and sunlight. Food may be insufficient in quantity, or in the variety of its nutritive constituents, or of such quality as to render it indigestible, and, in either case, the blood will become more and more defective in the proportion of its nutritive elements, and some of the tissues will be correspondingly impoverished.

There are but few persons in this country who suffer from inability to procure a sufficient amount of food. In the feeding and training of children, however, errors of much importance are frequent among all classes. Among the poor we often find large families living on the coarser and cheaper articles of food, with but little variety from week to week, and at the same time occupying damp, uncleanly, and ill-ventilated apartments; and glandular swellings, chronic ophthalmias, caries of the bones, and other local evidences of scrofulous and tubercular tendencies are common among them. Quite as often among the rich and fashionable we find the infants and young children committed to the care of nurses, kept much within the limits of the nursery, and indulged so freely in the use of saccharine matter, consisting of sugar, candies, sweet-meats, and sweet-cakes, at any and all times of the day, that they lose all relish for plain bread, milk, meat, and other nitrogenous food. The result is that they grow delicate, slender, thin in muscles, with narrow chests, unusual mental vivacity, and extreme susceptibility to all kinds of impressions. The anxious mothers always assert that they are so delicate they will take cold every time they are allowed to go out. It is among the children so trained that we find many of the best samples of the scrofulous constitutional condition, accompanied by frequent temporary, and sometimes permanent, enlargement of the lymphatic glands of the neck. And of those belonging to the same class who live beyond childhood and youth, there are many who become tuberculous between the ages of eighteen and thirty years.

Nothing is more certainly proved by abundant observation, than the fact that long continued living on food deficient in some of the elements needed for healthy growth and repair of living structure, is capable of modifying the assimilative processes in such a way as to develop imperfect cells and other tissue elements. And if to the use of food thus deficient, there be added living and sleeping in inadequately ventilated apartments, too little habitual exercise of the muscles of the chest and upper extremities, and clothing either inadequate for protection against sudden and extreme atmospheric changes or so adjusted as to impede the free expansion of the chest, you have a combination of influences, which, if long continued, are certain to so modify the properties of the blood and organized tissues of the body, as to establish some one of the special diatheses or morbid constitutional conditions, whether it be scrofulous, tuberculous, leucocythæmic, or rheumatic. It is also true, that a morbid constitutional condition thus acquired, if well established, is capable of being transmitted from parent to child, and thus start a new line of hereditary influence.

A somewhat careful study of the etiology of constitutional diseases, has led me to the conclusion that the habits of a people in regard to diet, drinks, dress, occupations, and the construction and cleanliness of houses, have far more to do with the production and propagation of the scrofulous, tuberculous, leucocythæmic, cancerous and gouty diatheses, than the elements and influences included under the head of climate; while the latter exert a controlling influence in the formation of the rheumatic predisposition. While the protracted influence of low temperature and dampness, combined with either impure air or insufficient food, tends strongly to produce the scrofulous and tuberculous affections; a climate characterized by low temperature with a high degree of moisture, and accompanied by frequent thermometric changes, without other bad influences, equally tends to create the rheumatic affections, by habitual interference with the natural exhalations from the cutaneous surface, and the consequent retention of certain acid constituents in excess in the blood. The excretory material thus retained evidently acts as an irritant, increasing the susceptibility of the fibrous tissues, and the plasticity of the blood, and thus placing the individual in the most favorable condition for the development of active rheumatic inflammation, with general fever from the temporary action of any exciting cause; or without any such intervention, by long continuance, inducing those slow hypertrophies and indurations of the fibrous and connective tissues, in different parts of the body, that constitute the purely chronic rheumatic affections so frequently met with in all cold, damp, and variable climates.

The most prominent characteristics of the climate in the whole Northern belt of our own country, from the eastern foot of the

Rocky mountains to the Atlantic coast, are long and very variable transition seasons (spring and autumn), with a predominance of cold and dampness. And it is exactly over this same belt of country that the population furnishes the highest ratio of the prevalence of rheumatic and catarrhal diseases, as shown many years since in the admirable work on the climate and diseases of the United States, by Dr. Samuel Forrey, formerly of the medical staff of the United States Army, and confirmed by the statistics of Dr. Daniel Drake, in the first volume of his work on the topography and diseases of the great interior valley of this continent. While it is true that the rheumatic diathesis is generally the result of habitual retention of excretory products capable of increasing the excitability of the organized tissues and plasticity of the blood, such retention is not always the result of unfavorable atmospheric or external impressions. On the contrary, I have seen numerous instances in which the same pathological conditio were reached, in some cases by protracted muscular exercise by which the products of tissue metamorphosis were developed faster than they could be eliminated through the natural channels; and in others, by such changes in habits or occupation that a previous habit of active out-door physical exercises sufficient to excite daily increased cutaneous exhalation, was exchanged for one of confinement or purely passive exercise, and consequently less activity in the cutaneous surface.

The gouty constitution or diathesis like the rheumatic, involves an increase of susceptibility in the fibrous or connective tissues, but with less plasticity of the blood, and more tendency to deficiency of the red corpuscles; and the causes most efficient in producing it, are such as directly lessen the action of oxygen in the natural processes of tissue metamorphosis, and the evolvement of those products that are eliminated by the kidneys, instead of the cutaneous structure. The presence of alcohol in the blood lessens the interchange of oxygen and carbonic acid gas through the lungs, and retards the molecular changes in the tissues; consequently its moderate daily use in the form of wine and other fermented drinks, keeps the blood in a state of imperfect decarbonization, diminishing the action of oxygen on the carbonaceous elements of the tissues, and favoring, first, fatty accumulations and subsequently, fatty degenerations. If the individual thus habitually using, moderately, alcoholic drinks, at the same time indulges the appetite for animal food, and takes very little muscular exercise, he will fail to eliminate the elements of urea, uric acid and the salts of sodium through the kidneys sufficiently fast to prevent the blood and tissues from retaining them in excess. It is the habitual presence of this excess of elements naturally excreted by the kidneys, in connection with the imperfect oxygenation and decarbonization of the blood, that induces those changes in the properties of the tissues which constitute the special gouty diathesis; and that every now and then cause the accumulation of such an amount of uric acid and urate of sodium as to excite the characteristic local nflammations of acute and chronic gout. In some instances the pathological conditions just mentioned as constituting the gouty diathesis, have been produced by habitual indulgence in the use of rich food, and the avoidance of all active physical exercise, without the use of either fermented or distilled liquors. But such cases are very rare, and so far as they have come under my observation there has been reason to suspect some degree of hereditary predisposition derived from the more remote ancestry.

*Pathological Inferences*—Fro [illegible] what I have now said in regard to causes capable of favoring the formation of constitutional diseases and their mode of action, you may deduce the following pathological conclusions:

First. That all the affections of this class involve, as a primary pathological condition, such a modification of the properties of the organized structures of the body as to render them morbidly susceptible to impressions, and to alter the molecular movements concerned in the processes of assimilation, nutrition and metamorphosis of tissues.

Second. The modification of properties just mentioned may result from hereditary transmission, or from the moderate but long continued action of such causes as are capable of either impairing the processes of assimilation and nutrition; or those of tissue metamorphosis and the excretion of waste products.

Third. In scrofula, tuberculosis, Addison's disease, and pernicious anæmia, the special modification of tissue properties is such as to increase the susceptibility by which the patients become morbidly sensitive or unduly influenced by almost every kind of external impression, and such an impairment of vital affinity that the formative processes by which the elements of tissues are evolved in the blood and attracted to their proper places in tissue growth and repair, are rendered imperfect and result in the formation of aplastic or cacoplastic material, as found in the caseous and tuberculous deposits and degenerations; or so greatly impaired as to arrest the formative processes altogether, as in the pernicious anæmia.

Fourth. In leucocythæmia and carcinoma there is less alteration of the susceptibility or excitability, but such an alteration of the vital affinity or force, controlling the formative processes as to result in an increase of the leucocytes and lymphoid cells, leading in the one disease to their marked excess in the blood, with hypertrophy of the adenoid glandular structures in different parts of the body, and in the other to a more specific and localized cell and fibrous development, constituting the varieties of cancerous tumors capable of development chiefly in the dermoid and glandular structures containing epithelium.

Fifth. In rheumatism or gout, the general diathesis or modification of tissue properties is such as to increase in a marked degree the susceptibility or general irritability of the organized structures, and to so modify the molecular movements in the metamorphic and excretory processes as to cause the retention and consequent accumulation in the blood, of an excess of certain excretory products, which, by their action on the already morbidly susceptible tissues, are capable of exciting the specific local inflammations of rheumatism and gout. In the present status of pathological investigations, I may state it as probable that the retained excretory products in rheumatism are chiefly lactic acid, and the lactic acid salts; and in gout, the uric acid and urates.

*Principles of Treatment.*—From the statements I have made concerning the causes and general pathology of the whole class of constitutional diseases, you will readily perceive that their practical management involves two distinct objects, namely: the removal of the general constitutional vice or predisposition, and the treatment of the various local affections that may appear from time to time during the progress of each individual case. The accomplishment of the first object will depend, mainly, on our ability to remove the patient from the further action of those causes and influences that favor the development of the particu-diathesis in question, and to substitute in their place such hygenic and sanitary measures as will bring a strong influence in the opposite direction. As all these diatheses, when not hereditary, are the result of influences acting moderately through long periods of time, so they can be removed only by influences acting with equal persistence in such direction as to induce an opposite effect. In all these affections, so far as the constitutional condition is concerned, a resort to active temporary medication of any kind, is both unphilosophical and useless. And yet, there are some medicines capable of affording material aid to the patient, if properly selected, given in moderate doses and continued for a long time. One of the chief difficulties in treating successfully all constitutional diseases and defects, is the inability of the patient and his friends to appreciate the necessity for persistence in the use of whatever remedial agents or influences are deemed necessary. It seems difficult for them, and sometimes even for the physician, to realize the fact that morbid conditions and processes which have been years in developing, or may have been inherited, cannot be removed or permanently corrected by the use of this or that remedy for a few days, or by a vacation from school or business, and a change of air, exercise, or climate, for a few weeks, or at most, a few months.

Consequently we see but few well devised and persistently executed plans of treatment adopted for either preventing or curing the constitutional conditions now under consideration. As a general rule you see the children of scrofulous, tuberculous, cancerous, syphilitic, and gouty parents, receiving no more attention in regard to their physical training than those of healthy parents. Yet it is during the period of childhood and youth, while the structures of the body are undergoing active development, that we have the best, if not the only, opportunity to correct such morbid tendencies as result from hereditary influence. And every physician should regard it as one of his most important professional duties to note the special morbid tendencies of all the families who rely upon him for medical services, and be as careful to point out the means for correcting them, as he is to prescribe medicines when they are actively sick. The family physician should realize that he is the guardian of the health of the families by whom he is employed, and he should so far interest himself in the welfare of the children especialy, that in his professional intercourse he should make such suggestions from time to time regarding the physical exercise, diet, dress, and education of the children as may be necessary to correct hereditary defects or acquired morbid tendencies during the years when such corrections are possible. I cannot too strongly impress upon each one of you the importance of this subject.

As the leading pathological, or morbid elements of the scrofulous and tuberculous diatheses are undue excitability, coincident with impairment of vital affinity or formative force, and possibly deficiency of the phosphatic and calcium compounds in the blood, so the remedial measures adopted should be such as are most efficient in lessening the former and in increasing the two latter. Among the most important of these measures is a plentiful supply of dry pure air, at a genial temperature for outdoor exercise or exposure; a sufficient quantity and variety of nutritious and easily digestible food; clothing of such quality and so adjusted as will best protect the cutaneous surface from sudden and severe atmospheric changes, and leave all the important movements and functions of the body free from mechanical interference; and such habitual daily muscular or physical exercises as tend to increase the development and strength of the muscular structures generally, and especialy those of the chest and upper extremities.

The heads of all families should be fully advised by their physician of the necessity of free ventilation in every part of their dwellings, and especially in their sleeping-rooms. Neither children nor adults should be allowed to sleep in cellar or basement rooms, or rooms anywhere that do not admit of free ventilation and sunlight, and afford, when closed, at least 800 cubic feet of space for each person occupying them. The physiological law, that regular habitual exercise, within certain limits, increases the amount and improves the quality of nutrition, is one of primary importance, as affording a means for correcting the defects and inequalities of development, whether hereditary or acquired, which exist in a large proportion of all the varieties of constitutional disease. By good air, a fair variety of good food, and regular daily exercise, weak and slender muscles can be made compact and strong; narrow chests with deficient air space can be made broader and more capacious; and with a more complete oxygenation and decarbonization of the blood will come healthier secretory actions and more perfect digestion, assimilation and nutrition. Thus, a bad constitution, or decided predisposition to disease, can be changed into one healthy, and even strong. But it requires much time, judicious direction, and undeviating steadiness of purpose in the daily execution of the work or play directed. And when the morbid conditions or defects have become well developed, and the period cf growth nearly or quite completed, before the systematic work of correction has been commenced, it may become necessary to add to the hygienic and sanitary measures already alluded to, a change of climate either temporary or permanent. As a general rule, you will find it most beneficial to send those who have been habitually living in interior valleys, of moist and alluvial formation, either to dry, mild, and elevated mountain ranges, or to the sea shore or on on sea voyages.

Those whose chief defects consist in slender muscles, narrow chests, and undue sensitivenesss of the respiratory organs, will do best in the mild, dry and pure air of the mountains; while those whose defects are chiefly in the digestive and assimilative organs will do best in the more stimulating and alterant atmosphere of the ocean. And yet those who have either inherited or acquired defects while permanently residing near the sea, will often be equally benefited by a change, either to the interior valleys or mountains.

If, in the management of the scrofulous, tuberculous, and kindred diatheses, medicinal agents are resorted to, they should be of such a nature as to be capable of diminishing the general excitability and of promoting the efficiency of the assimilative processes. In other words they should be soothing, tonic, and corrective, or mildly alterant. In former times small doses of the aqueous solution of iodine, given in some mildly sedative vegetable infusion soon after each meal-time, were much used and with good effect. The vegetable infusions most used were those of the sarsaparilla, prunus virginiana, cimicifuga racemosa, and pipsissewa. During the last fifteen or twenty years I have had frequent occasion to recommend for the same purposes a combination of two parts of the syrup of iodide of calcium with one part of the fluid extract of humulus lupulus, or hop. To patients over fifteen years of age, four cubic centimeters (fl. ʒi) of this mixture may be given just after each regular meal-time. To younger children the dose should be less. If the patient becomes weary of taking this, I substitute the syrup of lacto-phosphate of calcium for two or three weeks, after which the other can be resumed.

But in counteracting the constitutional predispositions now under consideration, no medicines, however long their use may be continued, can be relied upon to the exclusion or neglect of the hygienic and climatic influences to which I have referred.

The diathesis or constitutional condition favoring the development of the various forms of cancerous or malignant growths is one of the most obscure in the list of chronic general diseases. I am aware that many of the pathologists and practical surgeons of the present day regard all this class of morbid structural developments as primarily local, and claim that the general cachexia or diathesis is secondary, and the result of the diffusion of the cancer cells, or germs, originating in the local affection. But the fact that the predisposition to the disease is capable of hereditary transmission, while the local development of cancerous structure is often postponed until after the middle period of life, shows that there must be some deviation from the strictly healthy condition of the properties that govern the combinations of organic matter in the development of tissue elements, prior to the first germ of local morbid structure. Again, the fact that in a very large majority of cases cancerous growths re-appear after the earliest and most complete removal of the first unhealthy structure, points to the same conclusion. I have assumed, therefore, that there is a cancerous diathesis, or constitutional predisposition, which, if not removed, will, in due time, lead to the development of some variety of cancerous structure. In a large proportion of cases this diathesis is the result of hereditary influence; but that it may be acquired without such influence is also proved by the history of many cases in which no prior existence of this form of disease can be traced in either line of ancestry.

By what circumstances connected with diet, drinks, modes of living, or climate, the formation of such a diathesis is favored or counteracted, very little is known. In 1866 my colleague in the department of surgery, Dr. E. Andrews, by a careful and accurate examination of the mortality statistics of this country, as returned by the United States census for 1860, found a much higher ratio of deaths from cancerous diseases in the six New England States, and next in New York, Pennsylvania, New Jersey, and Delaware, the ratio steadily diminishing as he progressed south through the Atlantic States to the peninsula of Florida. The ratio was higher in the States occupying the northern part of the interior valley of the continent than in those farther south, the lowest ratio of all being in the extreme southwest, embracing the States of Texas, Missouri, Louisiana, Arkansas, and New Mexico. The last named State returned only one death from cancer, to two hundred and seventy from all diseases, while Vermont returned one from cancer to forty from all diseases. These figures would appear to show that the prevalence of cancerous affections was favored by a cold, variable, and damp climate, such as that which characterizes the northeastern and northern belt of the United States, and to be opposed by one that is mild and dry.

A more thorough examination of this part of the subject will probably demonstrate the proposition that cancerous affections prevail most wherever a cold and variable climate coexists with density of population, thereby following very nearly the same law of prevalence as scrofula and tuberculosis.* Many facts have come under my own observation favoring the idea that a liberal use of meat, coupled with in-door occupations, or sedentary habits, had a tendency to increase the cancerous predisposition, as it certainly does the local cancerous growths after they have com-

*See Relations of Cancer and Consumption to Climate in the United States. By E. Andrews, M. D. Chicago Medical Examiner, Vol. VII., p. 737. 1866.

menced. In the present state of medical knowledge, perhaps the best advice you can give to parties who, from known hereditary predisposition or otherwise, are desirous of counteracting the development of cancerous diseases in any of the structures of the body, is that they shall live in a mild, dry climate, remote from and elevated above the sea; to take free exercise in the open air; to use meat only sparingly; and wholly avoid all use of alcoholic drinks and tobacco. The principles that should govern us in the management of the rheumatic and gouty diatheses are plainly inferable from what I have already said regarding their mode of development; and the details of their application will be further explained when I come to speak of the active local developments of these affections. Having completed what I deem important to say regarding the general management of constitutional diseases, I shall reserve the consideration of the treatment necessary after local affections have become apparent, until I call your attention to each of the several diseases included in this group separately.

---

THE AGE OF GERMAN MEDICAL COLLEGES.—The university of Prague was founded anno Dei 1348; Vienna 1365; Heidelberg 1386; Leipzig 1409; Freiburg 1454; Greifswald 1456; Basel 1460; Munich 1472; Jena 1558. Wuerzburg, 1582; Göttingen 1737; Berlin 1810; Zurich 1833. Wuerzburg is celebrating this year its third centenary jubilee.

BERN (SWITZERLAND).—The law enforcing vaccination has been rejected by the popular vote. (There are some "cantons" in Switzerland which are just 500 years behind the present time, the dark shades arising from the mountains shortening the convolutions of the brains of the inhabitants).

PROF. GERMAIN SEE, the well-known French physiologist, has written an article (*L'Union Médicale*) on the effects of convallaria majalis, recommending the remedy highly in all diseases of the heart.

